# Rapid and Remarkable Response to Rituximab in a Case of Lupus Enteritis: Challenging the Norm

**DOI:** 10.31138/mjr.080624.rar

**Published:** 2024-06-08

**Authors:** Albader Hamza Hussein, Bayan Lafi Altamimi

**Affiliations:** Rheumatology Department, King Fahad Hospital, Madinah Al Munawwarah, Saudi Arabia

**Keywords:** lupus enteritis, systemic lupus erythematosus, Rituximab, response rate, case report

## Abstract

Lupus enteritis is a rare gastrointestinal complication of systemic lupus erythematosus (SLE) associated with significant morbidity and mortality. Rituximab, a monoclonal antibody targeting CD20-positive B cells, has shown promise in refractory SLE cases. We present a case of a 45-year-old female with SLE who developed lupus enteritis and experienced an unusually rapid and remarkable response to Rituximab. The patient presented with severe abdominal pain and distension. Within two days of Rituximab treatment, the patient’s abdominal pain, distension, and associated complications resolved completely. This exceptional response challenges the typical timeline of Rituximab efficacy in SLE and highlights the need for further investigation into the factors influencing treatment response. Understanding the mechanisms underlying such rapid improvement may provide insights into SLE pathogenesis and guide therapeutic strategies for optimal outcomes.

## INTRODUCTION

Rituximab is a chimeric monoclonal antibody that targets CD20-positive B lymphocytes, leading to their depletion and subsequent modulation of the immune response.^1,2^ It has been widely used in the treatment of various autoimmune disorders, including systemic lupus erythematosus (SLE) and its associated complications.^1^ SLE is a chronic autoimmune disease characterised by immune dysregulation and multi-organ involvement.^3,4^ Lupus nephritis, hematologic abnormalities, and serositis are common manifestations of SLE that can significantly impact patient outcomes.^3,4^

Rituximab has demonstrated efficacy in the management of SLE, particularly in cases refractory to conventional immunosuppressive therapies.^1,2,5^ It has been approved for the treatment of moderate-to-severe SLE with active lupus nephritis, and its off-label use has also shown promising results in the management of other SLE-related complications.^2,5^ The mechanism of action of Rituximab involves targeting and depleting CD20-positive B lymphocytes, which play a pivotal role in the pathogenesis of SLE.^1^ By reducing the B cell population, Rituximab effectively dampens the production of autoantibodies and attenuates the autoimmune response.^1,2^

While Rituximab has demonstrated therapeutic benefits in SLE, the response rate can vary among patients.^5^ The complete response rate to Rituximab in SLE is up to 30%, with a significant proportion of patients experiencing improvement in disease activity and organ involvement.^5^ However, the timeframe for clinical improvement can vary, and it typically takes several weeks to months to observe the full therapeutic effects of Rituximab.^5^

In this case report, we present an intriguing case of a patient with SLE who exhibited an exceptional and rapid response to Rituximab therapy. Despite the rarity and high mortality rate associated with lupus enteritis, our patient experienced a dramatic resolution of symptoms within two days of Rituximab administration. Lupus enteritis is a rare manifestation of SLE characterised by inflammation of the gastrointestinal tract, leading to abdominal pain, diarrhoea, and other gastrointestinal symptoms.^2,6^ The patient presented with severe abdominal pain, distension, and complications such as ascites and pleural effusion, which are often indicative of a poor prognosis.^6^ However, to our surprise, the patient’s symptoms and complications resolved rapidly, highlighting the remarkable efficacy of Rituximab in her case.

## CASE

We present a case of a 45-year-old female patient who has been suffering from SLE since 2016. The patient was diagnosed with SLE based on the EULAR/ACR criteria. She had positive serology for antinuclear antibodies (ANA) and for anti-dsDNA, low complement levels of C4 (0.134) shown by immunofluorescence assay (IFA), subnephrotic range proteinuria (0.75 g/day), inflammatory polyarthritis involving the fingers, wrists, and feet. Her signs and symptoms included unprovoked deep vein thrombosis (DVT), anaemia, and lymphopenia. She was maintained on Prednisone 5 mg per oral once daily, Azathioprine 100 mg per oral once daily, and Hydroxychloroquine 400 mg per oral once daily with good response.

On the 18th of October 2022, the patient presented to the emergency department (ER) with complaints of generalised abdominal pain, nausea, vomiting, and diarrhoea that persisted for ten days. She had previously been evaluated in another hospital and was treated with oral antibiotics as an outpatient for gastroenteritis. The CT scan of her abdomen showed mild proctosigmoiditis, and she was discharged with instructions to continue antibiotics. However, her symptoms did not improve, and she returned to the hospital after five days with worsening abdominal pain and distention.

**Figure 1.**
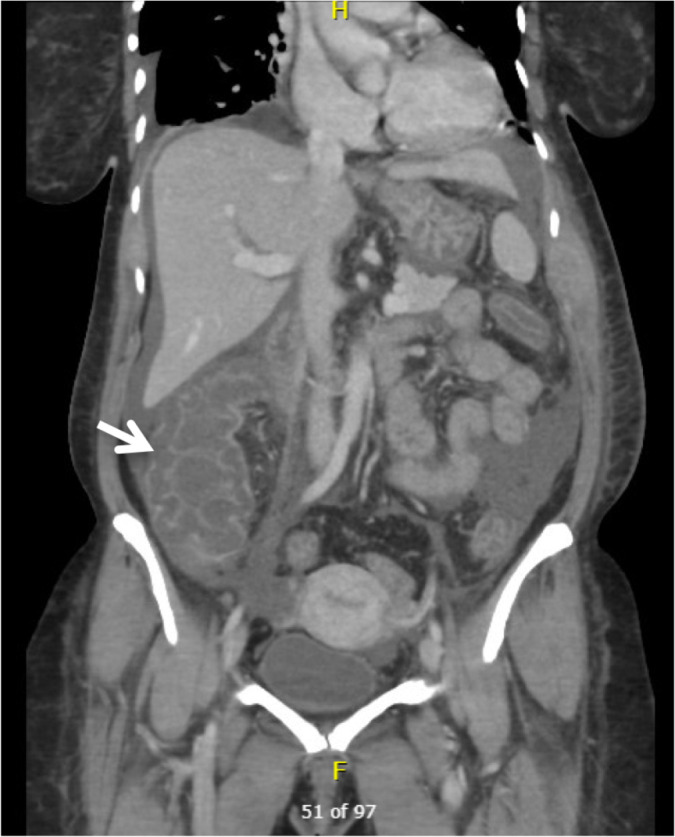
CT Angiogram of coronal view. The abdomen shows mural extensive thickening associated with submucosal oedema of colon (arrow).

Upon readmission on the 22nd of October 2022, a CT scan with intravenous (IV) contrast revealed extensive diffuse thickening of the colon and rectum walls with mild wall enhancement, suspecting ischemic colitis (**Figure 1**). The superior and inferior mesenteric arteries were patent. Small non-occlusive eccentric filling defects were noted at the main artery segments, representing mostly arteritis changes. A mild amount of free intra-abdominal fluid and a right pleural effusion was also seen. The patient was kept nil per os (NPO) and was administered IV Ceftriaxone, therapeutic anticoagulants, and IV Hydrocortisone 300 mg.

**Figure 2.**
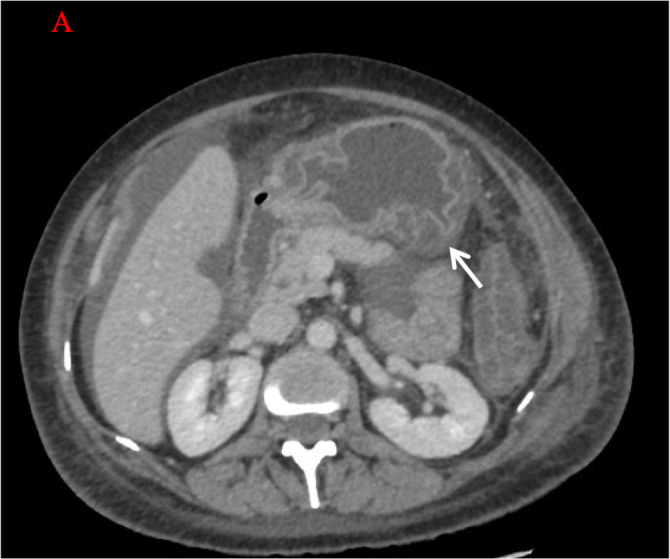
Contrast-enhanced CT of the abdomen images in axial **(A)** and coronal **(B)** plane demonstrating stomach wall oedema (arrow) and ascites.

**Figure 3.**
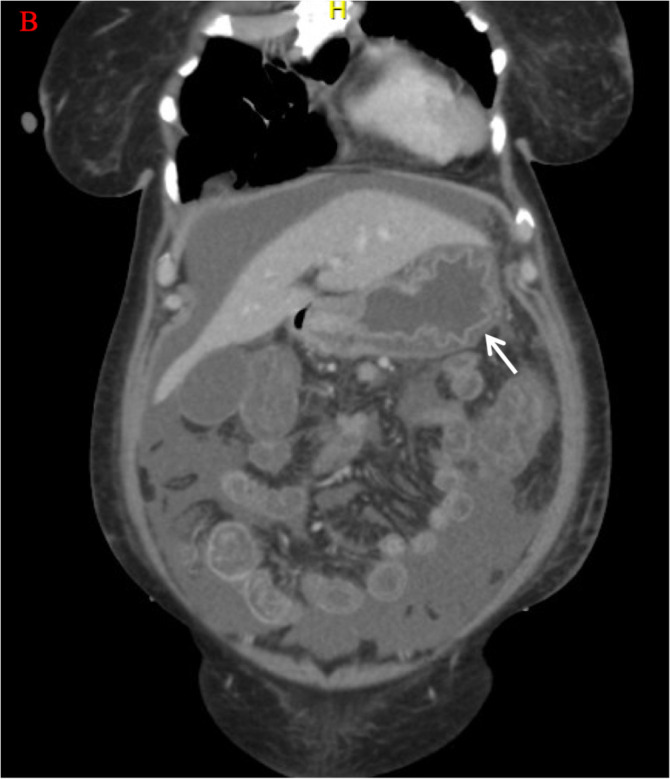
CT Angiogram abdomen: circumferential, symmetrical, multisegmented mural thickening with associated submucosal oedema (Target sign) seen (arrows).

**Figure 4.**
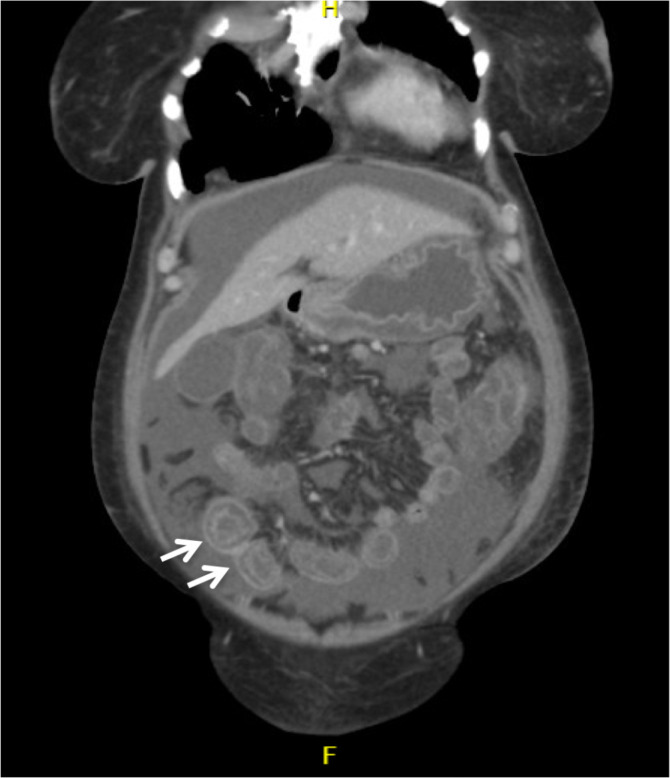
Axial CT Angiogram of the abdomen shows bilateral pleural effusion, right side more than left side (arrow).

Despite treatment, the patient’s symptoms did not improve, and she was referred to our hospital on 2nd November 2022 with a diagnosis of active SLE and bowel ischemia. She complained of generalised abdominal pain, nausea, and vomiting when presenting to our hospital. She was afebrile with a temperature of 37°C, and vital signs were within normal ranges, with a heart rate of 94 beats per minute, blood pressure of 139/89 mmHg, and oxygen saturation of 97%. Upon physical examination, the patient presented with severe abdominal tenderness and distension, with positive shifting dullness. There was no rash or synovitis present, and no organomegaly was observed. The patient’s bowel sounds were normal. Laboratory results showed normocytic anaemia (haemoglobin: 9.4 g/dl), lymphopenia (180 k/μL), hypocomplementemia (C3:0.45 g/l, C4: 0.08 g/l), and hypokalaemia (potassium: 2.9 mmol/l). CT angiography showed diffuse abnormalities in the gastrointestinal tract, including stomach and ileum wall thickening, submucosal oedema, ascites, and mesenteric fat stranding (**Figures 2 and 3**). Additionally, she had bilateral pleural effusion (**Figure 4**).

The patient was diagnosed with lupus enteritis, that lead to ischemic bowel, and serositis (pleural effusion, ascites). Her electrolytes were corrected. The surgical team recommended keeping her NPO, starting her on IV fluids, and keeping her under observation, with laparotomy only to be performed if her condition deteriorated. However, the patient’s status remained unchanged despite symptomatic treatment.

Azathioprine was stopped and the patient got started on pulse IV Methylprednisolone 1000 mg for three days and Rituximab 1 g (RA-protocol), and her clinical condition significantly improved on the second day of treatment. Her abdominal pain and distention resolved, and she had no further episodes of nausea or vomiting.

The patient was later transitioned to and discharged on Hydroxychloroquine 400 mg per oral once daily and Prednisolone 60 mg per oral daily with tapering. Two weeks later, the patient came back for the second dose of Rituximab, and she was symptoms-free. The prednisolone got tapered down to 50 mg per oral once daily and the patient was instructed to taper it down further 10 mg every ten days until she reaches 10 mg per oral once daily and continue for ten days. After that, Prednisolone was tapered down to 5 mg per oral once daily for ten days then was stopped. One month later, the patient visited the clinic for follow up. She was asymptomatic and did not have any complaints. She was started on Mycophenolate Mofetil 1 g per oral twice a day and was instructed to continue the discharge medications as instructed previously. The patient remained compliant to the administered medications and instructions. Evidently, at the three-month follow-up, she remained asymptomatic and her laboratory investigations (CBC, electrolytes, renal function tests, and urinalysis) remained within normal ranges. Since the patient remained clinically asymptomatic and her basic laboratory investigations were within normal ranges, she did not need to undergo follow up investigatory imaging. This case highlights the importance of considering lupus enteritis in patients with SLE presenting with abdominal pain, particularly if their symptoms do not improve with standard treatment. Additionally, using Rituximab may be a viable treatment option in such cases.

## DISCUSSION

The presented case describes a 45-year-old female patient with a longstanding history of systemic lupus erythematosus (SLE) who developed an uncommon and potentially life-threatening complication known as lupus enteritis. Lupus enteritis is a rare manifestation of SLE characterised by inflammation of the small bowel. It is associated with significant risks, including bowel ischemia, perforation, and mortality rates ranging from 8% to 14%.^6^ Prompt diagnosis and management are essential to optimise patient outcomes.^7^

The management of lupus enteritis typically involves a multifaceted approach aimed at controlling SLE disease activity and mitigating bowel inflammation. Treatment strategies include immunosuppressive agents, corticosteroids, and supportive care. Immunosuppressive medications, such as azathioprine, mycophenolate mofetil, or cyclophosphamide, are commonly employed to suppress the abnormal immune response in SLE and reduce overall disease activity.^2,8^ Corticosteroids, such as prednisone, are used to control inflammation and provide symptomatic relief.^8^ Additionally, supportive measures such as pain management, nutritional support, and close monitoring for complications are crucial aspects of patient care.^2,8^

Rituximab, a monoclonal antibody targeting CD20-positive B cells, has demonstrated efficacy in the treatment of refractory SLE. By depleting B cells, Rituximab reduces autoantibody production and modulates the dysregulated immune response observed in SLE.^1,2,5^ It has been shown to improve disease activity and reduce corticosteroid requirements in patients with refractory SLE.^2,5^ However, the response rate to Rituximab can vary among patients, with clinical improvement typically observed within weeks to months.^5^

In our case, the patient exhibited a remarkable and unexpectedly rapid response to Rituximab. Within just two days of initiating Rituximab treatment, the patient experienced a dramatic improvement in her symptoms, with complete resolution of abdominal pain, distention, and associated complications such as ascites. To the best of our knowledge, such a rapid and robust response to Rituximab in the context of lupus enteritis has not been extensively documented in the literature.

Several factors may contribute to the exceptional response observed in our patient. First, it is possible that the underlying disease process driving her lupus enteritis predominantly involved B-cell-mediated immune activation. Rituximab’s targeted mechanism of action, which specifically depletes B cells, could have led to a rapid reduction in autoantibody production and subsequent suppression of the inflammatory cascade. Second, the early initiation of Rituximab treatment in the course of lupus enteritis may have played a pivotal role in preventing further tissue damage and promoting rapid resolution of inflammation. Early intervention has been associated with better treatment outcomes in various autoimmune diseases, including SLE.^9^ Finally, individual variations in disease pathogenesis, genetic factors, or unique immunological profiles could also contribute to the observed rapid response to Rituximab in this case.

While there is limited literature documenting cases of lupus enteritis specifically treated with Rituximab, there have been reports of successful use of Rituximab in the management of refractory SLE.^10^ These cases support the notion that Rituximab can induce rapid and significant clinical improvements, including the resolution of gastrointestinal manifestations.^11^ Further studies and a larger number of reported cases are necessary to better understand the mechanisms underlying such a dramatic response and to establish Rituximab as a potential therapeutic option for lupus enteritis.

In summary, this case highlights the exceptional and rapid response to Rituximab in a patient with lupus enteritis and active SLE. The prompt resolution of symptoms within two days of initiating Rituximab treatment is unusual, considering the typically slower response rate observed with this medication. Further investigation is warranted to elucidate the factors contributing to this rapid response and to evaluate the efficacy and safety of Rituximab in the management of lupus enteritis. This case underscores the need for heightened awareness of lupus enteritis as a potential complication in SLE patients presenting with abdominal symptoms and emphasises the potential role of Rituximab in the management of this challenging condition.

## PATIENT PERSPECTIVE

Experiencing the sudden onset of severe abdominal pain was a frightening situation for the patient, especially given her history with SLE. The uncertainty surrounding the diagnosis was overwhelming, and the pain seemed unbearable. However, the rapid response to Rituximab was astonishing. Within just two days, the pain and discomfort that had burdened her for a while began to improve. This turn of events filled her with hope and gratitude. It was a profound relief to see the treatment take effect swiftly. The patient was committed to her recovery, attending all follow-up appointments with a sense of determination and gratitude. The attentive care she received, coupled with the remarkable effectiveness of the treatment, restored not only her physical well-being but also her confidence in the medical journey ahead. She is grateful for the expertise and compassion of the healthcare team that guided her through this challenging period.

## CONCLUSION

In conclusion, our case report describes a patient with SLE who developed lupus enteritis and exhibited a remarkably rapid and complete response to Rituximab treatment. This finding suggests that Rituximab, with its ability to target CD20-positive B cells, may hold promise as a therapeutic option for lupus enteritis. The exceptional response observed highlights the need for further research and larger studies to explore the efficacy and safety of Rituximab in this context. The case emphasises the importance of considering lupus enteritis in SLE patients presenting with abdominal symptoms and underscores the need for early recognition and appropriate management. Further investigations are warranted to identify predictive factors and optimize treatment strategies for patients with lupus enteritis.

## References

[1] Cartron G Blasco H Paintaud G Watier H Le Guellec C. Pharmacokinetics of rituximab and its clinical use: Thought for the best use? Crit Rev Oncol Hematol [Internet] 2007 Apr 1;62(1):43–52. Available from: https://www.sciencedirect.com/science/article/abs/pii/S1040842806002009 17287129 10.1016/j.critrevonc.2006.09.004

[2] Janssens P Arnaud L Galicier L Mathian A Hie M Sene D Lupus enteritis: from clinical findings to therapeutic management. Orphanet J Rare Dis [Internet]. 2013 May 3 [cited 2020 Oct 2];8:67. Available from: https://www.ncbi.nlm.nih.gov/pmc/articles/PMC3651279/10.1186/1750-1172-8-67 PMC3651279 23642042

[3] Manson JJ Isenberg DA. The pathogenesis of systemic lupus erythematosus. Neth J Med [Internet]. 2003 Nov 1;61(11):343–6. Available from: https://pubmed.ncbi.nlm.nih.gov/14768716/14768716

[4] Catalina MD Owen KA Labonte AC Grammer AC Lipsky PE. The pathogenesis of systemic lupus erythematosus: Harnessing big data to understand the molecular basis of lupus. J Autoimmun 2020 Jun;110:102359. 31806421 10.1016/j.jaut.2019.102359

[5] Díaz-Lagares Cándido Croca S Sangle Shirish Vital EM Catapano F Martinez-Berriotxoa A Efficacy of rituximab in 164 patients with biopsy-proven lupus nephritis: Pooled data from European cohorts. Autoimmun Rev 2012 Mar 1;11(5):357–64. 22032879 10.1016/j.autrev.2011.10.009

[6] Hoffman BI Katz WA. The gastrointestinal manifestations of systemic lupus erythematosus: A review of the literature. Semin Arthritis Rheum 1980 May;9(4):237–47. 6996096 10.1016/0049-0172(80)90016-5

[7] Tian XP. Gastrointestinal involvement in systemic lupus erythematosus: Insight into pathogenesis, diagnosis and treatment. World Journal of Gastroenterology. 2010;16(24):2971. 20572299 10.3748/wjg.v16.i24.2971 PMC2890936

[8] Lee C-K. Acute abdominal pain in systemic lupus erythematosus: focus on lupus enteritis (gastrointestinal vasculitis). Ann Rheum Dis 2002 Jun 1;61(6):547–50. 12006332 10.1136/ard.61.6.547 PMC1754133

[9] Ezeonyeji AN Isenberg DA. Early treatment with rituximab in newly diagnosed systemic lupus erythematosus patients: a steroid-sparing regimen. Rheumatology 2011 Nov 16;51(3):476–81. 22096015 10.1093/rheumatology/ker337

[10] Tanaka Y Nakayamada Shingo Yamaoka K Ohmura Koichiro Yasuda S. Rituximab in the real-world treatment of lupus nephritis: A retrospective cohort study in Japan. Mod Rheumatol 2022 Feb 15 [cited 2023 May 25];33(1):145–53. Available from: https://academic.oup.com/mr/article/33/1/145/6528634 10.1093/mr/roac007 35165714

[11] Kenar G Atay K Yüksek GE Öz B Koca SS. Gastrointestinal vasculitis due to systemic lupus erythematosus treated with rituximab: a case report. Lupus 2020 Mar 18;29(6):640–3. 32188302 10.1177/0961203320910803

